# EUS-guided drainage using hepaticocolostomy after esogastrectomy

**DOI:** 10.1016/j.vgie.2020.11.011

**Published:** 2020-12-13

**Authors:** Abdellah Hedjoudje, Bénédicte Jaïs, Alain Aubert, Pierre Cattan, Frédéric Prat

**Affiliations:** 1 Service d’endoscopie digestive, Hôpital Beaujon, Assistance publique des hôpitaux de Paris, Paris, France; 2 Service de Chirurgie générale, Digestive et Endocrinienne, Hôpital Saint Louis, Assistance publique des hôpitaux de Paris, Paris, France

A 72-year-old man with a malignant hilar biliary obstruction was referred to our endoscopy unit for biliary drainage. The patient had undergone esogastrectomy for an esophagogastric junction adenocarcinoma in 2011, followed by total gastrectomy with colon interposition in 2019 for a gastric adenocarcinoma. There was an esophagocolonic anastomosis and a side-to-side duodenocolonic anastomosis. The patient was referred for biliary obstruction due to perihilar carcinomatosis with no sign of cholangitis but with dysphagia related to an upper anastomosis stenosis.

After balloon dilation through a stenotic esocolonic anastomosis, a large-channel linear-array EUS endoscope was used to puncture the left intrahepatic bile duct through the coloplasty with a 19-gauge access needle and a 0.035-inch straight-type guidewire (Fig. 1). Subsequently, we attempted to pass the guidewire across the confluence to bridge the left and right hepatic ducts. The hilar stricture was dilated before application of electrocautery throughout the colohepatic tract using a 6F cystostome (Fig. 2). Finally, a 10-cm × 10-mm partially covered self-expandable metallic stent was deployed (Fig. 3A and B).

**Figure 1:**
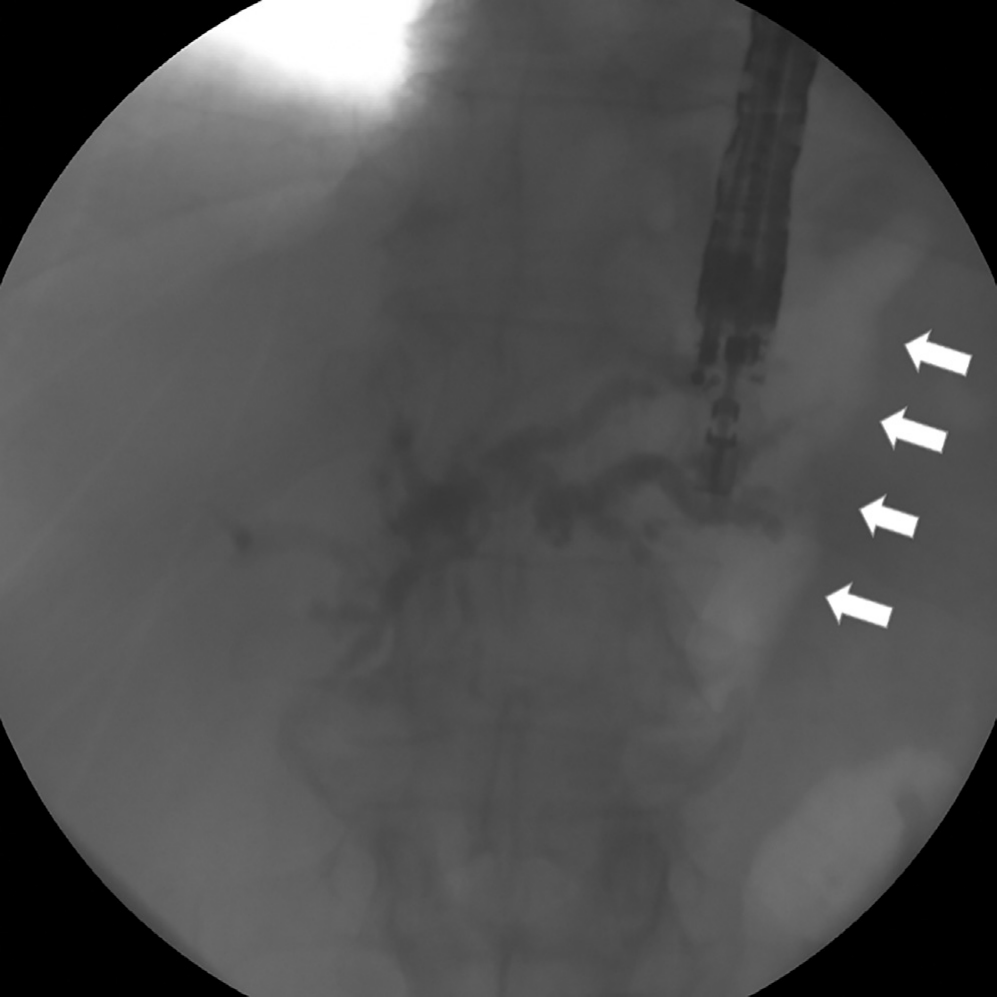
EUS-guided cholangiography after puncture through the coloplasty. *Arrows* show air inside the coloplasty. Contrast injection showed limited hilar involvement with some passage to the right hepatic ducts, whereas the common bile duct was obstructed over its full length up to the superior biliary confluen.

**Figure 2:**
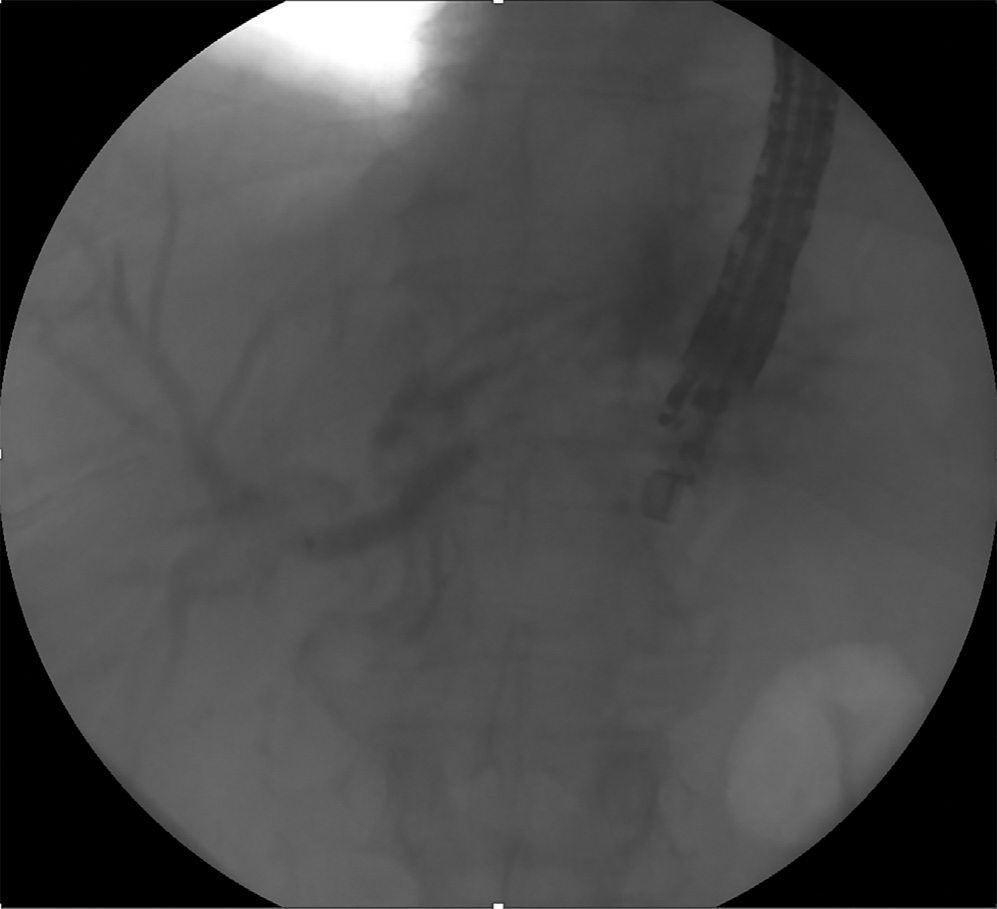
Balloon dilation of the hilar stricture.

**Figure 3:**
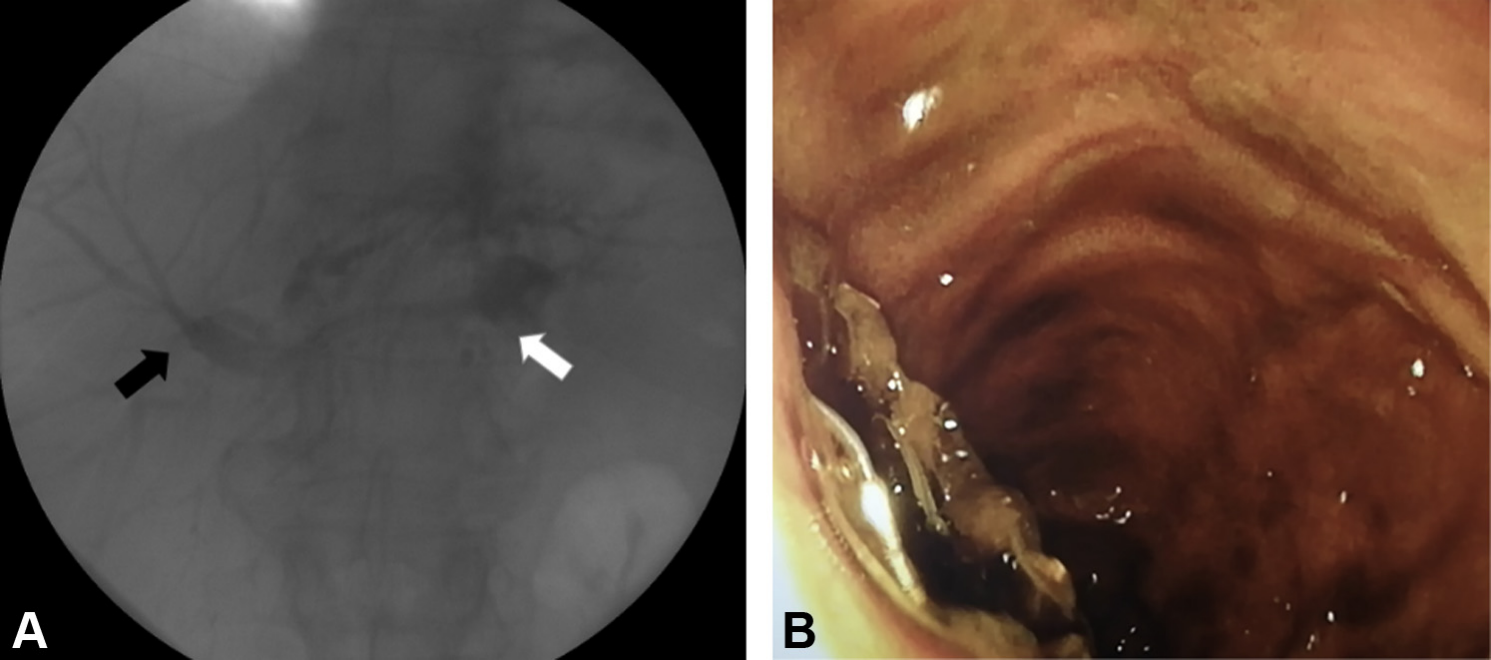
Fluoroscopic and endoscopic images of the hepaticocolonic stent after release. **A,***Arrows* showing distal (right hepatic, *black*) and proximal (luminal, *white*) ends of the biliary stent. **B,** Protrusion of the large covered metallic flange from the colonic wall.

The patient recovered uneventfully, with liver test improvements 3 days after the procedure (total bilirubin 62 vs 120 μM [minus 50%], alkaline phosphatase 298 vs 494 IU/L, and gamma-Glutamyl transpeptidase 119 vs 219 IU/L). CT of the abdomen showed widespread pneumobilia (Video 1, available online at www.giejournal.org). To our knowledge, this is the first report of transcolonic biliary drainage, showing that this rare procedure can be performed using the same steps as regular EUS-guided hepaticogastrostomy.

## Disclosure

*All authors disclosed no financial relationships.*

## Supplementary data

Video 1 Video showing the extent of the hepaticocolonic stent 5 days after release.

